# Revalidation of the Argentinian pouched lamprey *Geotria macrostoma* (Burmeister, 1868) with molecular and morphological evidence

**DOI:** 10.1371/journal.pone.0233792

**Published:** 2020-05-29

**Authors:** Carla Riva-Rossi, Diego Andrés Barrasso, Cindy Baker, Analía Pamela Quiroga, Claudio Baigún, Néstor Guillermo Basso

**Affiliations:** 1 Instituto de Diversidad y Evolución Austral (IDEAus-CONICET), Puerto Madryn, Chubut, Argentina; 2 Sección Herpetología, División Zoología Vertebrados, Facultad de Ciencias Naturales y Museo, Universidad Nacional de La Plata (UNLP), La Plata, Buenos Aires, Argentina; 3 National Institute of Water & Atmospheric Research Ltd (NIWA), Hamilton, New Zealand; 4 Instituto de Investigación e Ingeniería Ambiental (CONICET-UNSAM), San Martín, Buenos Aires, Argentina; University of Georgia, UNITED STATES

## Abstract

**Background:**

The Argentinian pouched lamprey, classified as *Petromyzon macrostomus* Burmeister, 1868 was first described in 1867 in De La Plata River, in Buenos Aires, Argentina, and subsequently recorded in several rivers from Patagonia. Since its original description, the validity of *P*. *macrostomus* was questioned by several ichthyologists and 36 years after its original discovery it was considered a junior synonym of *Geotria australis* Gray, 1851. For a long time, the taxonomic status of *G*. *australis* has been uncertain, largely due to the misinterpretations of the morphological alterations that occur during sexual maturation, including the arrangement of teeth, size and position of fins and cloaca, and the development of an exceptionally large gular pouch in males. In this study, the taxonomic status of *Geotria* from across the “species” range was evaluated using both molecular analysis and examination of morphological characteristics.

**Methodology/principal findings:**

Phylogenetic and species delimitation analyses based on mitochondrial DNA sequences of Cytochrome b (*Cyt b*) and Cytochrome C Oxidase Subunit 1 (*COI*) genes, along with morphological analysis of diagnostic characters reported in the original descriptions of the species were used to assess genetic and morphological variation within *Geotria* and to determine the specific status of the Argentinian lamprey. These analyses revealed that *Geotria* from Argentina constitutes a well differentiated lineage from Chilean and Australasian populations. The position of the cloaca and the distance between the second dorsal and caudal fins in sub-adult individuals, and at previous life stages, can be used to distinguish between the two species. In addition, the genetic distance between *G*. *macrostoma* and *G*. *australis* for the *COI* and *Cyt b* mitochondrial genes is higher than both intra- and inter-specific distances reported for other Petromyzontiformes.

**Conclusions/significance:**

Our results indicate that the Argentinian pouched lamprey, found along a broad latitudinal gradient on the south-west Atlantic coast of South America, should be named as *Geotria macrostoma* (Burmeister, 1868) and not as *G*. *australis* Gray 1851, returning to its earliest valid designation in Argentina. *Geotria macrostoma* can now be considered as the single lamprey species inhabiting Argentinian Patagonia, with distinct local adaptations and evolutionary potential. It is essential that this distinctiveness is recognized in order to guide future conservation and management actions against imminent threats posed by human actions in the major basins of Patagonia.

## Introduction

Lampreys are jawless fishes representing one of the most ancient groups of vertebrates, which have oftentimes been called “living fossils” because of the resemblance of some morphological features to those found in early fossils from the Devonian period (360 million years ago) [1–4]. Extant lampreys possess several distinct morphological features, such as a round mouth (“cyclostomes”), a piston cartilage, horny epidermal teeth on the suctorial disc, and seven gill openings on each side of the body [5]. These jawless fishes belong to the Order Petromyzontiformes, a group with an antitropical distribution, containing 41 species widely distributed in the Northern and Southern Hemispheres [5–7]. The only exceptions to this distribution is the genus *Tetrapleurodon* that occurs in high altitude streams at latitude 20º N [7]. In the Northern Hemisphere, there is only one lamprey family (Petromyzontidae) including eight genera containing 37 of the 41 species, while in the Southern Hemisphere there are two poorly diversified families (Geotriidae and Mordaciidae) each of them with one genus comprising, respectively, one and three species [5–9].

The life cycle of anadromous lampreys, those that migrate to the ocean to feed and return to freshwater to breed, begins in freshwater with a larval phase; these larval lampreys are filter-feeders and live buried in the silt and sand within rivers. At the end of the larval period (3 to 4 years) [10], the larvae metamorphose and become downstream-migrating juveniles and migrate to the ocean where they feed parasitically on fishes blood and body tissues. When the young adults are fully grown at sea, they cease feeding and return to freshwater as sub-adults (3 to 4 years), where they become sexually mature, spawn and then die [10]. For most species reaching sexual maturity and spawning occurs within several months upon re-entry to freshwater habitats, but *G*. *australis* and *Entosphenus tridentatus* Pacific lamprey display a protracted maturation phase, where spawning occurs after 12 to 16 months in freshwater [11].

Although nine species of lampreys exhibit this anadromous and parasitic life cycle, others do not migrate to the sea but remain resident in freshwater feeding parasitically, while others reach sexual maturity in freshwater without a juvenile feeding period (“nonparasitic”) [2, 6, 7, 12, 13]. In some genera of lampreys, parasitic anadromous vs. nonparasitic freshwater species are called “paired species”. Since they are morphologically and, in many cases, genetically similar, it is assumed that the freshwater nonparasitic species have evolved from a closely related parasitic form [5, 14, 15]. Nonparasitic forms are found in most lamprey genera, except for *Petromyzon*, *Caspiomyzon*, and *Geotria* [8].

The genus *Geotria* has been considered to contain a single species, *Geotria australis* Gray 1851 [16], which occurs throughout New Zealand, southern and western Australia, Tasmania, Chile and Argentina, including the Malvinas (Falkland) Islands. The taxonomic status of *Geotria* still remains unresolved, largely due to the misinterpretation of the morphological changes that occur during its sexual maturation [8, 17–19]. These changes affect body size, the number and arrangement of the teeth, the height and position of the dorsal and caudal fins, the size of oral disk relative to the head, and the development of an exceptionally large gular pouch in males [8, 18, 20, 21]. Based on this morphological variation, several taxonomic rearrangements and nomenclatural acts have been proposed, but all of them are now considered as *Geotria australis* [5].

**Fig 1:**
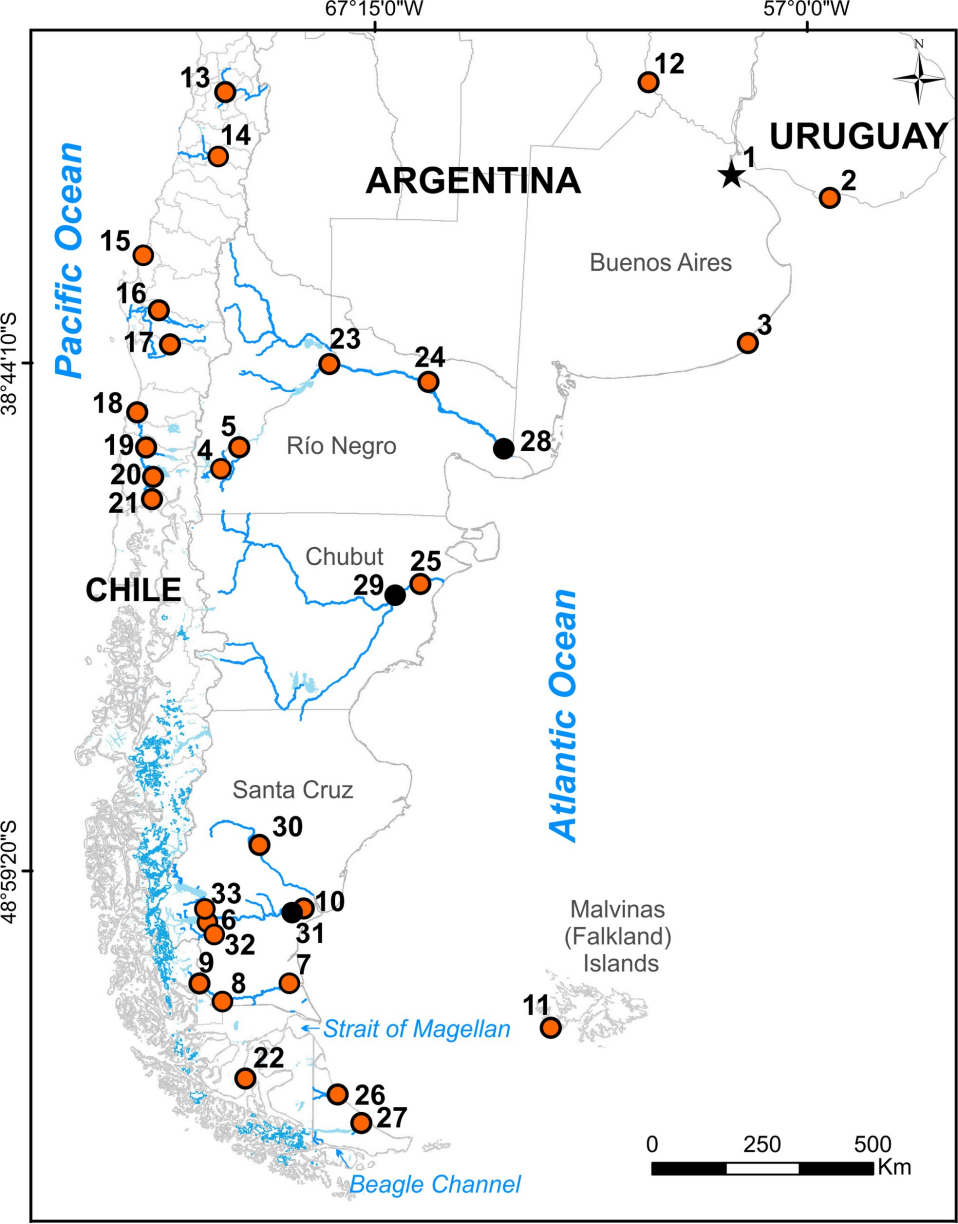
Map of southern South America showing sampling locations and published records of *G*. *australis* in Argentina, Chile, and Uruguay. Main river basins and the Patagonian Ice Sheets, with extent of contemporary glaciers shown in blue. The black star indicates the type locality of *Petromyzon macrostomus* Burmeister from de la Plata River, orange circles indicate bibliographic records of *G*. *australis*, and black circles indicate our collection sites. References: 1, de la Plata River (Argentina, [22–24, 26]); 2, Isla de Flores (Uruguay, [23, 26]); 3, Mar del Plata (Argentina, [25, 38]); 4, Nahuel Huapi Lake (Argentina, [25]); 5, Nuevo Lake (Argentina, [25, 38]); 6, 32, Argentino Lake (Argentina, [25, 26, 29]); 7, Gallegos River (Argentina, [30]); 8, Rubens River (Argentina, [30]); 9, Turbio River (Argentina, [30, 41]); 10, Santa Cruz River (Argentina, [33]); 11, Malvinas (Falkland) Islands (Argentina, [34]); 12, Paraná River (Argentina, [38]); 13, Santiago (Chile, [48]); 14, Canal del Molino (Chile, [48]); 15, Bahía Concepción (Chile, [48]); 16, Angol (Chile, [48]); 17, Puren River (Chile, [48]); 18, Valdivia River (Chile, [48]); 19, Osorno (Chile, [48]); 20, Lake Llanquihue (Chile, [48]); 21, Maullín River (Chile, [48]); 22, San Juan River (Chile, [37]); 23 Plottier, Negro River Argentina, [39]); 24, Choele Choel Island (Argentina, [39]); 25, Trelew City, Chubut River (Argentina, [39]); 26, Grande River (Argentina, [41]); 27, Fuego River (Argentina, [38]); 28, Negro River (Argentina, this study); 29, Chubut River (Argentina, this study); 30, Chico River (Argentina, this study); 31, Piedra Buena City (Argentina, this study); 33, La Leona River (Argentina, [40]).

In Argentina, lampreys were first recognized by Burmeister [22], who described a specimen collected in a street of Buenos Aires city in 1867 and named it *Petromyzon macrostomus*. In 1893, Berg [23] added information to Burmeister’s description and described a new individual collected at the island of Flores (Fig 1), near to Montevideo city (Uruguay) and proposed the new combination *Geotria macrostoma* (Burmeister, 1868). In subsequent years, Berg [24–26] described additional sub-adult and adult individuals collected from Buenos Aires city and from several waterbodies of Patagonia (see Fig 1), and proposed the name *Exomegas macrostomus* (following Gill’s nomenclature [27]) for those forms with a well-developed gular pouch, while he classified those forms without the gular pouch as *Geotria chilensis* [24, 26, 28]. In 1896, Lista [29] described an individual with an enlarged gular pouch from the Argentino Lake and, following Berg [26], called it also *Exomegas macrostomus* [29], Later, Smitt [30] described a distinct specimen of lamprey without the gular pouch from the Gallegos River and attributed the specimen to *Geotria macrostoma* var. *gallegensis* (Fig 1).

In 1909, Eigenmann [31] revised the taxonomic history of Chilean and Argentinian lampreys and recognized the occurrence of different species in Argentina: *G*. *chilensis* (de la Plata River), *Exomegas macrostomus* (de la Plata River, Patagonia), and *Exomegas macrostomus* var. *gallegensis* (Patagonia). Subsequently, Regan [32] only accepted the validity of *Geotria macrostoma* for Argentinian waters. In 1915, Lahille [33] described two species of Argentinian lampreys (“lamprea argentina”) in Argentina; *G*. *australis* (replacing *Exomegas macrostomus*) distributed from the Gallegos River to de la Plata River, and *G*. *chilensis* from the Santa Cruz River to the la Plata River.

However, in 1929, Maskell [17] compared specimens of *G*. *australis* from New Zealand with morphological descriptions available in the published records from Smitt [30] and Lahille [33], rejecting Lahille‘s *G*. *chilensis*, which he concluded was simply a sub-adult of *G*. *australis*. Based on his comparisons, Maskell [17] recognized only one valid species for the genus, *Geotria australis*, occurring throughout New Zealand, Australia, Tasmania, and South America. In 1937, Norman [34], revalidated *G*. *australis* as the only species within *Geotria* but warned that a direct comparison of individuals at all life stages between South America, Australia and New Zealand was still pending. In 1950, Nani [34] adopted the species designation recommended by Maskell [17] and Norman [34] and adopted the name *Geotria australis* for all the Argentinian lampreys. Despite this consensus, other authors continued to mention the occurrence of *Exomegas macrostomus* in South America [18, 35, 36]. The most recent mention of *Exomegas* corresponded to Sielfeld [37] who recorded one individual in the San Juan River (Chile) at the Pacific outlet of the Strait of Magellan (Fig 1) and designated it as *Exomegas macrostomus*.

Presently, scientific records of *G*. *australis* from Buenos Aires, Mar del Plata and Montevideo, already rare at the beginning of the 1900s (no more than 10 individuals; [38]), are almost non-existent. However, the species is widely distributed in several large Atlantic watersheds from Patagonia, such as Limay, Negro, Chubut, Santa Cruz, Gallegos and the Grande rivers [38–40], as well as in small streams from Tierra del Fuego [41] (Fig 1). It has also been recorded in coastal waters of the Malvinas (Falkland) Islands [34, 42] (Fig 1), however, the failure to corroborate these old records has led to the conclusion that *G*. *australis* may enter the Malvinas (Falkland) Islands waters occasionally, through straying from its migratory route between South America and South Georgia [7, 43].

Recent studies based on morphological data found great differences between representatives of *Geotria* from Argentina and those from Australasia and Chile [5, 20, 21, 44], and recently these differences have been corroborated with genetic data by Nardi [41] who reported a different species of *Geotria* inhabiting rivers in Patagonia, at the southern tip of Argentina [41]. In the present study, the recent collection of downstream migrating juveniles and sub-adult lampreys from the largest Atlantic basins from Patagonia allowed us to verify these morphological and genetic divergence reported in these previous studies and, based on this information, determine whether one or two species of *Geotria* exist in Argentina [e.g., 33]. For this purpose, we evaluated the occurrence of distinct species within the genus *Geotria* under the “phylogenetic species concept” [45–47]. Therefore, we used two mitochondrial DNA markers to reconstruct the phylogenetic relationships within *Geotria* and used this information to discuss its evolutionary biogeography and to evaluate the taxonomic status of Argentinian lampreys. To assess the specific designations, we reevaluate diagnostic characters proposed in original descriptions of the species and its synonyms across Argentina, Chile and Australasia.

## Materials and methods

### Sample collection

This study was carried out in accordance to the ethical regulations of CONICET (Consejo Nacional de Investigaciones Científicas y Tecnológicas) for biomedical and biological research with laboratory and farm animals and those obtained in nature (Resolution D 1047 Annex II of the year 2005). Fish capture and handling procedures were approved by specific permits issued by the Ministerio de Agricultura, Ganaderia y Pesca from the Río Negro Province (Resolution 007), by the Instituto Provincial del Agua, Administración General de Recursos Hídricos from the Chubut Province (Resolution 24/19DGAguas-IPA) and by the Ministerio de Producción, Comercio e Industria, Subsecretaría de Coordinación Pesquera from the Santa Cruz Province (Resolution MPCI 438818/18 del Provincia de Santa Cruz). Fish anesthesia and euthanasia was performed using a mild dose (30 mg/mL) and an overdose (100 mg/mL) of benzocaine (Parafarm, CABA, Argentina).

Between February and March 2019, 125 sub-adults were collected using fyke-nets in the lower Santa Cruz River (50.05°S, 69.01°W) during their upstream migration. In May 2019, 39 sub-adults from the lower Chubut River (43.45°S, 65.91°W) were collected by electrofishing and by hand during their upstream migration. Finally, in July 2019, three downstream migrating juveniles were collected by electrofishing in the lower Negro River (40.57°S, 63.56°W). For all fish captured, they were anesthetized and their external characters were examined. In addition, tissue samples were collected from fresh specimens for molecular analysis. A sample of 28 individuals (15 from the Santa Cruz River, 10 from the Chubut River, and the three juveniles from the Negro River) were euthanized with an overdose of benzocaine, stored at –20°C, and transported to the laboratory. These specimens were fixed in 10% neutral buffered formalin and deposited in the Ichthyiology Collection of the Instituto de Diversidad y Evolución Austral (IDEAus-CONICET), Puerto Madryn, Argentina (vouchers CNPICT2019/1 to CNPICT2019/28). The remaining fish were released back into the river after examination.

### Morphological analysis

**Fig 2:**
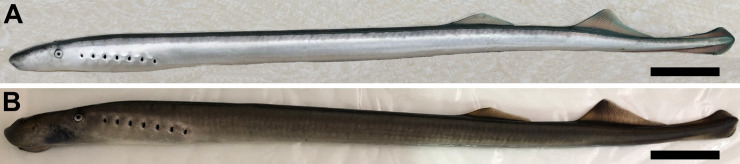
Argentinian lamprey from the Santa Cruz River examined in this study. A) Immature upstream migrant. B) Maturing sub-adult. Both individuals were tagged and released back into the river after examination. Scale bar = 5 cm.

For morphological analysis, the 125 sub-adult individuals collected at the Santa Cruz River and the 39 sub-adult individuals collected at the Chubut River were anesthetized, photographed on their left side, and examined for the main external characters cited as diagnostic in the literature of *Geotria* taxonomy (e.g., the position of the cloaca and the distance between the second dorsal and caudal fins in sub-adult individuals) [6, 7, 18, 20, 21] (Fig 2). Since the status of type specimens of *Petromyzon macrostomus* or *Geotria macrostoma* is unknown (collected specimens were deposited at the Museo Argentino de Ciencias Naturales–MACN—but holotypes were not designated), the state of these characters in fresh specimens were matched and confirmed with those reported in the original descriptions of the Argentinian lamprey provided by Burmeister [22], Berg [23, 25, 26], Smitt [30], Lahille [33], Sielfeld [37] and also with morphological descriptions of *Geotria australis* from Chile [20], New Zealand [17], and Australia [6].

For morphological comparisons with fresh specimens of *G*. *australis* we also obtained sub-adult lampreys from the Waikawa River, Southland, New Zealand (46.59°S 169.14°E,) during their upriver migration. Upstream migrant sub-adults (N = 300) were collected during August 2019 by hand from a rock weir located immediately above the tidal zone in the river. Sub-adults from the Waikawa River ranged in total length from 438 to 623 mm and at the time of capture they exhibited a dark brown coloration in body and fins. A sample of these lampreys (N = 83) were anesthetized, photographed on their left side, and examined for external morphometric characters.

### DNA extraction, amplification, sequencing and analysis

For molecular analysis, muscle tissue was taken from 23 individuals: 10 sub-adults from the Santa Cruz River, 10 sub-adults from the Chubut River, and three juveniles from the Negro River. Total genomic DNA was isolated using salt-extraction protocols [49] from muscle tissues stored in 96% ethanol at -20°C.

We amplified two mitochondrial markers, Cytochrome C Oxidase Subunit 1 (*COI*) and Cytochrome b (*Cyt b*). To amplified the *COI* fragment we used two primers designed for amphibians but with universal tails T3-AnF1 (5’‒AATAA CCCTC ACTAA AGACH AAYCA YAAAG AYATY GG‒3’) and AnR1 (5’‒AATAC GACTC ACTAT AGCCR AARAA TCARA ADARR TGTTG‒3’) following the thermal profile of the Polymerase Chain Reaction (PCR) proposed by the authors [50]: 3 min at 95ºC of initial denaturation, 35 cycles of 30 s at 94°C, 20 s of annealing at 50°C, and 1 min at 60°C of extension, followed by a final extension of 5 min at 60°C. For eight of the samples, three from Santa Cruz River, three from Negro River, and two from Chubut River, we also amplified a fragment of the *Cyt b* gene. For this we used the primers MVZ15 (5’‒GAACT AATGG CCCAC ACWWT ACGNA A‒3’; [51]) with addition of universal tail T3 and CB3-3’ (5’‒GGCAA ATAGG AARTA TCATT C‒3’; [52]), both widely used in several vertebrate groups. The PCR profile consisted of 2 min at 94°C of initial denaturation, 40 cycles of 30 s at 94°C, 45 s of annealing at 47°C, and 2 min at 72°C of extension, followed by a final extension of 6 min at 72°C. The PCR-products (~ 750 bp for *COI* and ~900 for *Cyt b*) were purified and sequenced in both directions at Macrogen Inc (Seoul, South Korea). Contigs for isolates sequenced were assembled using DNA Baser software (Heracle Biosoft, Pitesti, Romania). The obtained DNA sequences were registered at GenBank (see S1 Table).

Because our primers were not specific to lampreys, in the first step we aligned the fragment obtained for *COI* and *Cyt b* with complete genes extracted from the mitochondrial genome of *Geotria australis*, *Petromyzon marinus*, and *Ichthyomyzon unicuspis* (GenBank accession No. KT185629, U11880, KM267717), the alignments were runs in MAFFT online service using the G-INS-1 strategy [53].

### Genetic diversity and phylogenetic analyses

DNA fragments (*Cyt b*, and *COI*) were aligned with Clustal W [54], run in BioEdit [55] under default parameters. The number of haplotypes (Ht), singletons (Hs), haplotype diversity (Hd), and nucleotide diversity (π) were calculated for each gene using the DnaSP 6 software [56]. Uncorrected *p-distance*s were estimated employing the software MEGA 10 [47].

Phylogenetic relationships were reconstructed under the Maximum Parsimony Criterion (MP) and maximum likelihood (ML). For this purpose, we used as outgroups one Actinopterygii (*Amia calva*) and three Mixinidae (*Mixine glutinosa*, *Eptatretus burger*, and *Eptatretus atami*). Since the monophyly of Petomyzontidae, the Northern Hemisphere lampreys, has been tested already with morphology and molecular data [8, 44, 57] we selected only some species of this family. We included 23 *COI* and 8 *Cyt b* fragments generated by us from three Argentinian populations of *Geotria*, and all available GenBank sequences for Southern Hemisphere lampreys: Mordaciidae (*Mordacia lapicida*, *M*. *mordax*, and *M*. *precox*) and Geotriidae (four *Cyt b* sequences of Argentinian lampreys from the Turbio River, a tributary from the Gallegos River, and Grande River, recently published by Nardi et al. [41] and 18 *Geotria australis* from across its range). For several of the species included in our analysis the complete mitochondrial genome was available, thus for these we extracted the *Cyt b* and *COI* genes. In the species for which the complete genome was not available, we concatenated *Cyt b* and *COI* sequences with different accession number but originated at the same location. GenBank Accession Numbers, vouchers and sample locations are shown in S1 Table.

For MP analysis we used the software TNT [58], using a traditional search under default parameters, and swapping the trees with Tree Bisection-Reconnection (TBR). A strict consensus was calculated using all the most parsimonious trees found. Branch support was evaluated with 10,000 pseudoreplicates of jackknife [59] under default TNT settings, using 0.36 of removal probability. For ML analysis we used RAxML-HPC [60] using the GTRGAMMA model, with 100 heuristic searches and 1,000 bootstrap replicates. The analysis was run on the CIPRESS Science Gateway website [61]. Analyses were performed for each marker separately and combining the two genes in a single matrix. In both trees the species *Amia calva*, *Mixine glutinosa*, *Eptatretus burger*, and *Eptatretus atami* were used as outgroups. Additionally, for *Geotria*, we also constructed a haplotype Median Joining Network using Network v10 [62].

We evaluated all *Geotria* sequences under the ‘species delimitation’ concept, using Automatic Barcode Gap Discovery (ABGD) [53]. This method seeks to find “barcode gaps” by comparing pairwise differences among all sequences that discriminate inter- and intraspecific diversity. This approach was chosen because it does not require a phylogenetic framework. Analyses were run for the separated *Cyt b* and *COI* genes. The uncorrected *p-distances* (SD), Jukes-Cantor (JC69), and Kimura (K80) distances were used as nucleotide substitution models within each matrix. All analyses were conducted under default parameters (10 recursive steps, gap width of 1.5 and intraspecific divergence values between 0.001 and 0.1) through the ABGD web- server (http://wwwabi.snv.jussieu.fr/public/abgd/).

## Results

### Taxonomy

*Geotria macrostoma* (Burmeister, 1868)

*Petromyzon macrostomus* Burmeister, 1868: xxxvi [22]. Holotype: not designated [allegedly housed at the MACN]. Type locality: Buenos Aires, Argentina, collected at September 26, 1867.

*Geotria macrostoma* (Burmeister) Berg, 1893: p. 3–6, pl. 2. Redescription [23].

*Exomegas macrostomus* Gill, 1882, p. 524 [27]; Berg, 1895: 4 [25]; 1899: 91 [26]. Locality: Montevideo, Uruguay, de la Plata River and Argentino Lake, Argentina.

*Geotria chilensis* (Gray) Günther, 1870: 506 [28]; Berg, 1895: 121 [25]. Locality: Buenos Aires, Argentina.

*Geotria macrostoma gallegensis* Smitt, 1901: 26, pl. 4 [30]. Type locality: Gallegos River and tributaries, Ruben and Rio Turbio Rivers.

*Dionisia patagonica* Lahille, 1915: 374 [33]. Name erected on p. 374, and synonymized with *Geotria chilensis* (Gray) on p. 380. Distribution: from de la Plata River to the Santa Cruz River, Argentina.

*Geotria australis* Gray, 1851: 142, pl. 1 [16]. Type locality: Inkar Pinki R., Hobson's Bay or Onkaparinga, South Australia. Lahille [33] proposed it as synonym senior of *Exomegas macrostomus* and *Geotria macrostoma* on page 372. Distribution: Australia and Tasmania, Chile, New Zealand, and Argentina. Not mentioned from Argentina in the original description.

#### Distribution of *G*. *macrostoma* in South America

De la Plata River, Uruguay; de la Plata River to Tierra del Fuego, Argentina; Malvinas (Falkland) Islands, South Georgia. There is an isolated record of a spawning adult collected at the San Juan River (53°S), at the Chilean side of the Magellan Strait.

**Vernacular names in Argentina and Uruguay.** Lamprea de bolsa, lamprea argentina, bandera argentina.

**Fig 3:**
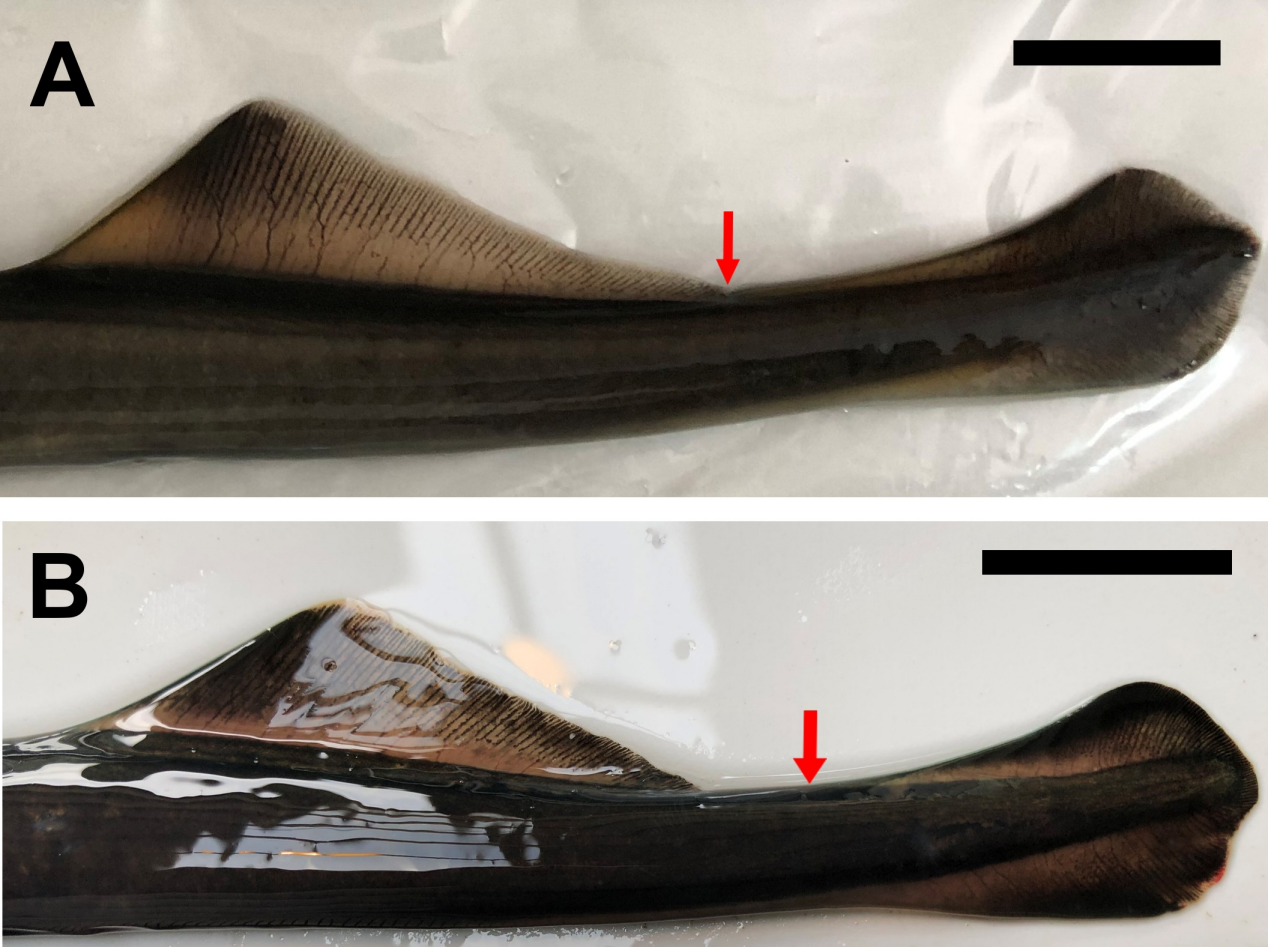
Position of the second dorsal and caudal fins in sub-adults of *Geotria*. A) Argentinian lamprey (Santa Cruz River). B) *Geotria australis* (Waikawa River). The red arrow indicates the origin of the caudal fin. Scale bar = 2 cm.

**Fig 4:**
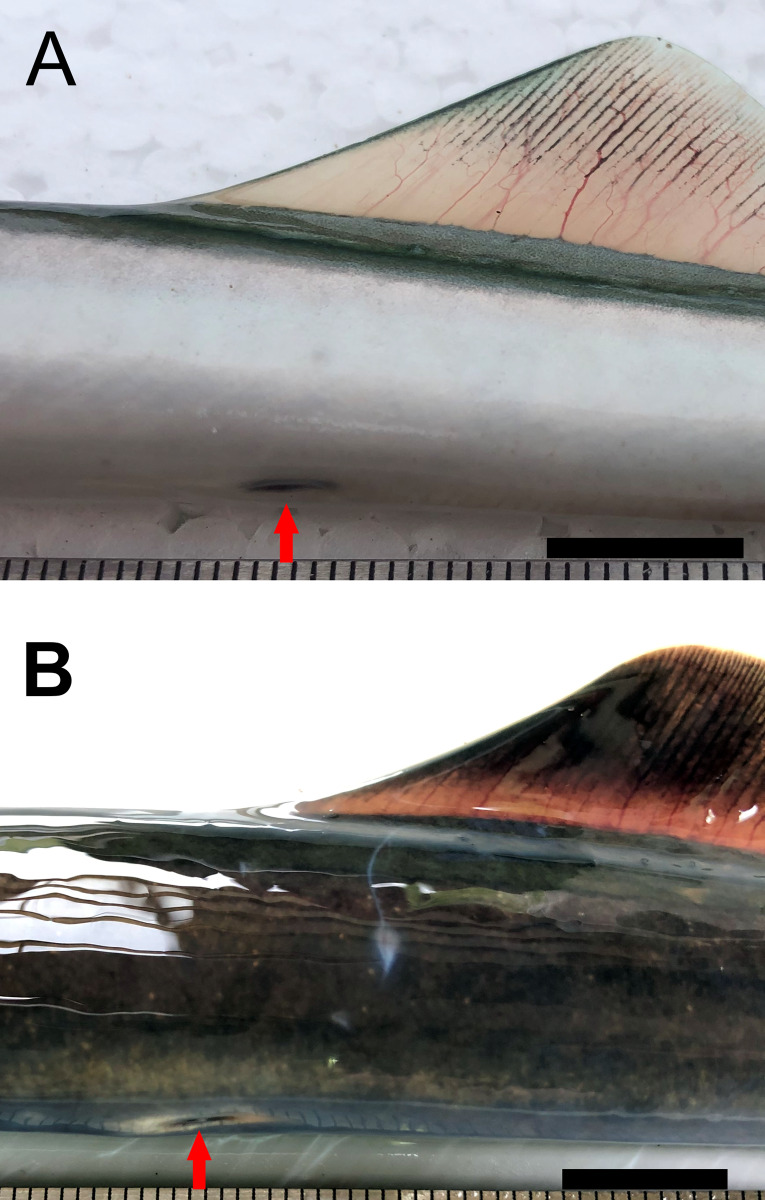
Position of the cloaca in sub-adults of *Geotria*. A) Argentinian lamprey (Santa Cruz River). B) *Geotria australis* (Waikawa River). The red arrow indicates the position of the cloaca. Scale bar = 1 cm. Adult lampreys caught in the Santa Cruz (Riva Rossi et al. unpublished data) and Negro Rivers (by local fishermen) showed dark brown body and fins and a well-developed gular pouch was observed in two mature males. In these fish the second dorsal and caudal fins are contiguous and the cloaca is positioned posterior to the origin of the second dorsal fin (Fig 5).

**Fig 5:**
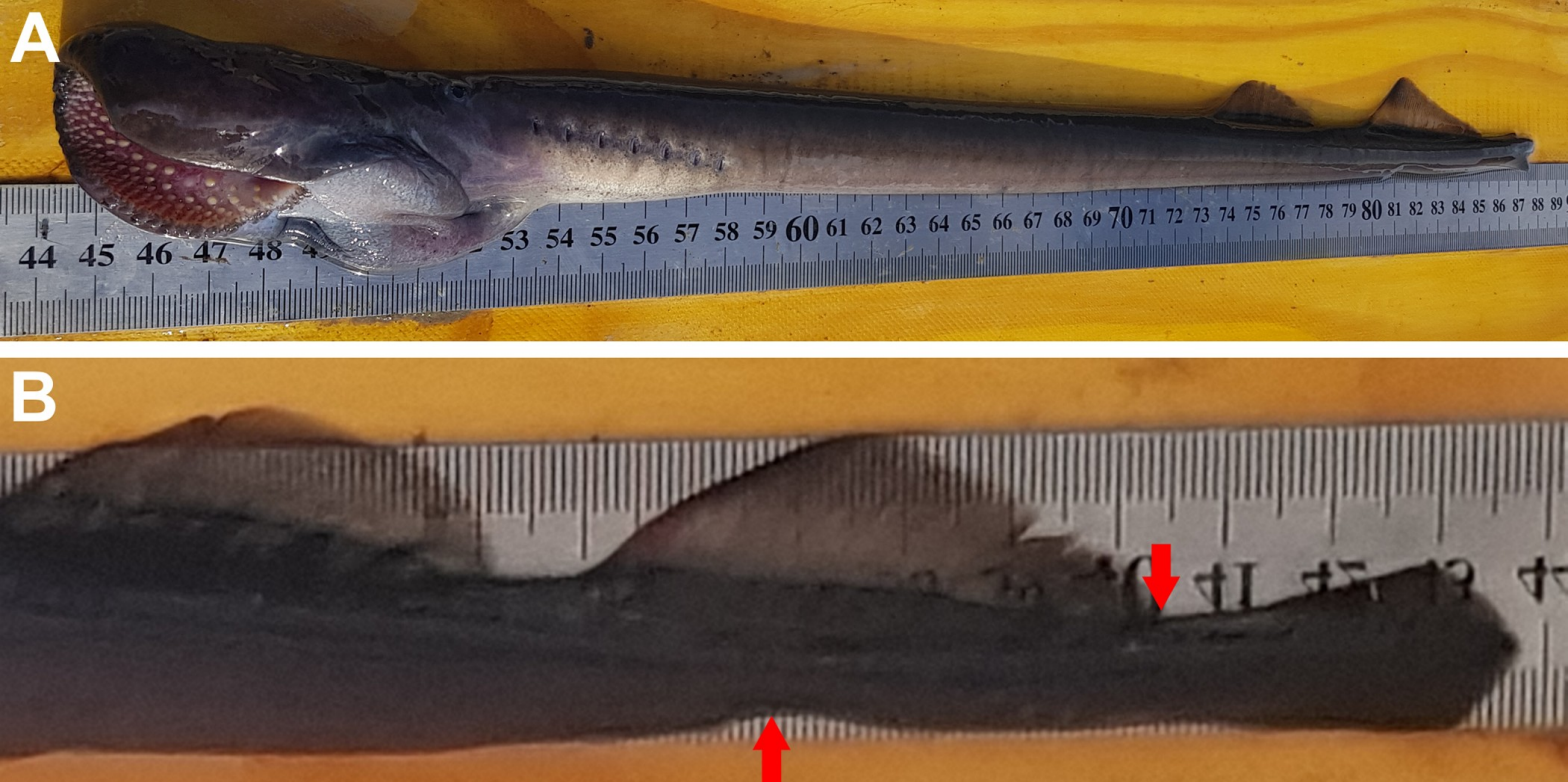
Adult lamprey from the Santa Cruz River. A) Mature male individual with its tail missing. B) Detail of the position of the second dorsal and caudal fins and the cloaca. The red arrow shows the origin of caudal fin and the position of the cloaca.

**Diagnosis.** *Geotria macrostoma* is distinguished from *G*. *australis* by the presence of a second dorsal fin connected with the caudal fin by a low skin fold and by the position of the cloaca posterior to the origin of the second dorsal fin in immature and mature adults [8, 22, 23, 25, 26, 29, 30, 33, 37] (Table 1, Figs 2, 3 and 4). In *G*. *australis* the second dorsal fin is separate from the caudal fin (Table 1, Fig 3) and the cloaca anterior to or under the origin of the second dorsal fin (Table 1, Fig 4) [6, 7, 16, 17, 20, 63]. While the position of fins and cloaca of mature *G*. *macrostoma* remains similar to that of juvenile and sub-adults (Fig 5), in a recent study Potter et al. [93] has shown that in spawning adults of *G*. *australis* the separation between the second dorsal fin and the caudal fin becomes reduced to a notch (Fig 2C of Potter et al. [93]), a condition that has never been described in previous studies [6, 7, 16, 17, 20, 63].

**Table 1:** Species, record location, and selected morphological characters to distinguish *Geotria australis* and Argentinian *Geotria*.

Author	Country of Record	Species	Stage	Second dorsal to caudal fin	Position of the cloaca	Reference
Gray 1851	Australia	*Geotria australis*	Adult	Separate ^a^	Not indicated	[16]
Burmeister 1868	Argentina	*Petromyzon macrostomus*	Adult	Not indicated	Posterior to the origin of the 2nd dorsal fin	[22]
Gunther 1870	Australia	*Geotria australis (on Gray’s holotype)*	Adult	Separate	Not indicated	[28]
Berg 1893	Argentina	*Geotria macrostoma (On Burmeister’s material)*	Adult	Close ^b^	Posterior to the origin of the 2nd dorsal fin	[23]
Berg 1893	Uruguay	*Geotria macrostoma*	Adult	Missing tail	Posterior to the origin of the 2nd dorsal fin ^c^	[23]
Berg 1895	Argentina	*Geotria chilensis*	Sub-adult	Contiguous	Posterior to the origin of the 2nd dorsal fin ^d^	[26]
Lista 1896	Argentina	*Exomegas macrostomus*	Adult	Contiguous	Posterior to the origin of the 2nd dorsal fin	[29]
Ogilby 1896	Australia	*Geotria australis*	Sub-adult /Adult	Separate	Below the origin of the 2nd dorsal fin	[63]
Berg 1899	Argentina	*Exomegas macrostomus*	Adult	Contiguous	Below the origin of the 2^nd^ dorsal fin	[25]
Smitt 1901	Argentina	*Geotria macrostoma var*. *galleguensis*	Adult	Contiguous	Posterior to the origin of the 2nd dorsal fin ^e^	[30]
Lahille 1915	Argentina	*Geotria chilensi*	Sub-adult	Distant ^f^	Posterior to the origin of the 2nd dorsal fin	[33]
Lahille 1915	Argentina	*Geotria australis*	Adult	Contiguous ^f^	Posterior to the origin of the 2nd dorsal fin	[33]
Maskell 1929	New Zealand	*Geotria australis*	Sub-adult	Separate	Below the origin of the 2nd dorsal fin ^g^	[17]
Maskell 1929	New Zealand	*Geotria australis*	Adult	Close	Possibly below the origin of the 2nd dorsal fin ^h^	[17]
De Buen 1961	Chile	*Geotria australis*	Adult	Separate	Not indicated	[64]
Potter 1986	Australia	*Geotria australis*	Sub-adult	Separate	Below the origin of the 2nd dorsal fin ^i^	[6]
Potter 1986	Australia	*Geotria australis*	Adult	Separate	Below the origin of the 2nd dorsal fin ^i^	[6]
Siefeld 1976	Chile, Strait of Magellan	*Exomegas macrostomus*	Adult	Contiguous	Posterior to the origin of the 2nd dorsal fin	[37]
Neira 1984	Chile	*Geotria australis*	Sub-adult	Separate	Under the origin of the 2nd dorsal fin	[20]
Neira 1984	Chile	*Geotria australis*	Adult	Separate	Under the origin of the 2nd dorsal fin	[20]
Gill et al 2003	Australasia. Chile, Argentina	*Geotria australis*	Sub-adult / adult		Anterior to or under the origin of the 2nd dorsal fin	[8]
Renaud 2011	Australasia. Chile, Argentina	*Geotria australis*	Sub-adult /Adult	Separate	Anterior to or under the origin of the 2nd dorsal fin; under the anterior half of the 2nd dorsal fin in individuals from Argentina	[7]
Potter et al	Australia	*Geotria australis*	Adult	Contiguous	Under the origin of the 2nd dorsal fin ^j^	[93]
Riva Rossi et al	Argentina	*Geotria sp*.	Sub-adult	Contiguous	Posterior to the origin of the 2nd dorsal fin	
Riva Rossi et al	Argentina	*Geotria sp*.	Adult	Contiguous	Posterior to the origin of the 2nd dorsal fin	
Riva Rossi et al	New Zealand	*Geotria australis*	Sub-adult	Separate	Anterior to or under the origin of the 2nd dorsal fin	

^a^ Shown in Plate V [16]

^b^ Position of the upper lobe of the caudal fin [23].

^C^ Shown in Plate 2 [23].

^d^ Shown in Plate 2 and Fig 2 [26].

^e^ Indicated in the Table of page 28 [30].

^f^ Position of the upper lobe of the caudal fin [33].

^g^ Shown in Fig 22 and indicated in the Table of page 191 [17].

^h^ Shown in Fig 23 in page 192 [17].

^i^ Shown in Fig 1 in page 11 [6].

^j^ Shown in Fig 2C [93].

#### Morphological description of *G*. *macrosotma*

In agreement with the original description of Burmeister [22], the redescription provided by Berg [23], and additional descriptions provided by others authors [25, 26, 28, 29, 30, 33, 37, 39], the sub-adult individuals of *G*. *macrostoma* revised in this study (total length from 412 to 629 millimeters) (Fig 2) presented a dark, enlarged oral papillae on each side of the oral disk, a supraoral lamina with four cusps (two pointed central ones flanked by broader lateral flanges), lingual teeth bi or tricuspid, two longitudinal lingual laminae, each with four unicuspid teeth, and one dark oral papilla on either side of the oral disc is enlarged. Two dorsal fins separate, the second dorsal and caudal fins contiguous and connected by a low skin fold in immature and mature adults (Table 1, Fig 3), and cloaca posterior to the origin of the second dorsal fin (Table 1, Fig 4). Adult males are characterized by the presence of a large gular pouch behind the head (Fig 5) and bicuspid lingual teeth [22, 23, 24, 25, 29, 30, 33, 37]. In the three downstream migrating juveniles from the Negro River the cloaca was also located posterior to the origin of the second dorsal fin.

### Genetic diversity and phylogenetic analyses

We obtained a fragment of 652 bp that aligned perfectly with the entire *COI* gene (~1557 bp) of *Geotria australis*, *Petromyzon marinus*, and *Ichthyomyzon unicuspis*, overlapping from bp 51 to 702 without ‘indels’. This fragment is similar to other *COI* fragments obtained with primers developed for fishes and used for lampreys [48]. For the *Cyt b* gene we obtained a fragment of 737 bp that aligned with the complete *Cyt b* gene (~1191 bp) of *G*. *australis*, *P*. *marinus*, and *I*. *unicuspis*, overlapping from bp 26 to 762 without ‘indels’ and with the *Cyt b* sequences of the Argentinian *Geotria* reported by Nardi et al [41], overlapping from bp 258 to 762 without ‘indels’.

**Fig 6:**
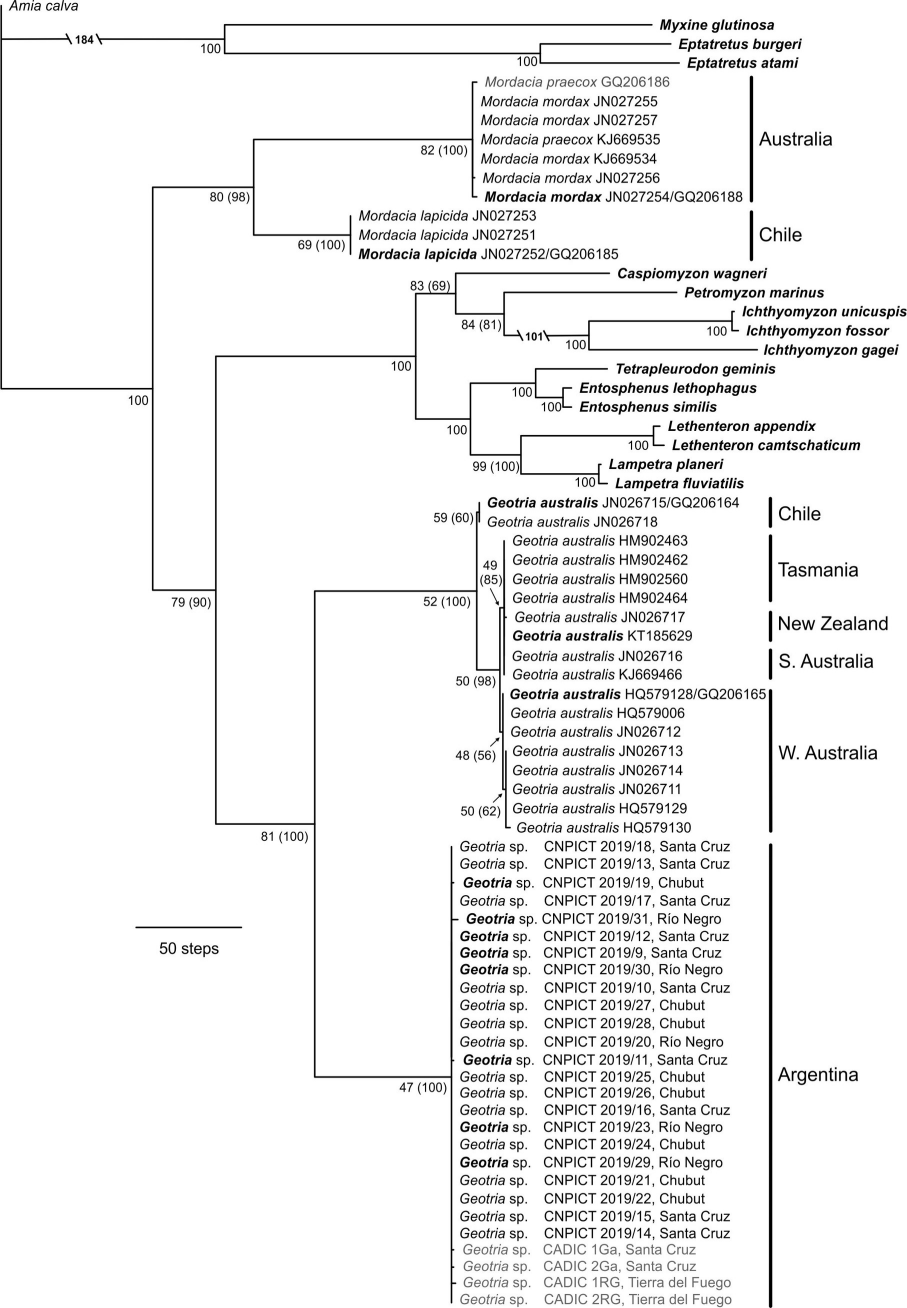
Strict consensus tree obtained from Maximum Parsimony analysis of the two mitochondrial markers. Strict consensus of 100 most parsimonious trees of 4130 steps. Branch lengths are proportional to parsimony transformations steps. Gaps were considered as fifth state. Name of samples for Argentinian *Geotria* are indicated by the institutional acronym and location (Province) of each sample. Terminal taxa where *COI* and *Cyt b* sequences were concatenated are indicated in bold, terminal taxa represented only by the *COI* fragment are shown in plain font and taxa represented only by the *Cyt b* fragment are shown in grey (see S1 Table). Numbers below the nodes indicate parsimony jackknife support. Values in parentheses show the support obtained when samples represented only by the *Cyt b* were excluded from the analysis (grey samples).

The MP analysis of the combined dataset produced 90 most parsimonious trees of 4071 steps. The strict consensus tree recovered three main clades within Petromyzontiformes: (A) Geotriidae, (B) Petromyzontidae, and (C) Mordaciidae, consistent with the current taxonomy of the group. In this tree, *Geotria australis* conformed as a well-supported monophyletic group, sister to the Northern Hemisphere lampreys (Petromyzontidae), while *Mordacia* was nested outside all other living petromyzontiforms (Fig 6). In the genus *Geotria*, all specimens from Argentina were recovered as an “Atlantic” clade with a high jackknife support value, sister to a well-supported “Pacific” clade, formed by specimens of *Geotria australis* from Chile and Australasia (when samples represented only by the *Cyt b* were excluded from analysis) (Fig 6). The Pacific clade was further subdivided into two groups containing on one side samples from Chile and, on the other, samples from Australasia. In this last group, haplotypes from Southern Australia, Tasmania and New Zealand were more closely related to each other than to haplotypes sampled in Western Australia.

Within the genus *Mordacia*, the Australian species (*M*. *praecox* and *M*. *mordax*) were recovered together with low divergence between them, but with high divergence from the Chilean *Mordacia lapicida*. In agreement with Gill [8] and Lang [44], all Northern Hemisphere lampreys (Petromyzontidae) were placed in a well-supported clade. The major lineages comprising this clade are a) *Lampetra*, *Entosphenus*, *Lethentheron* and *Tetrapleurodon;* and b) *Caspiomyzon*, *Petromyzon* and *Ichthyomyzon*. Both groups with high support. The ML tree of the combined dataset recovered almost the same topology obtained with MP analysis, only differing in the *Caspiomyzon* position within Petromizontidae (S1 Fig).

Genetic diversity for Argentinian *Geotria* sequences generated by us was extremely low, both for *Cyt b* (N = 8, Ht = 4, Hs = 3, Hd = 0.634 ± 0.184; π = 0.00102 ± 0.00036) and for the *COI* gene (N = 23, Ht = 2, Hs = 2, Hd = 0.087 ± 0.078; π = 0.00029 ± 0.00026), while higher genetic diversity was found in the *Geotria* clade from Australasia and Chile for both the *Cyt b* (Ht = 3, Hs = 15, Hd = 1.0 ± 0.272;π = 0.01357 ± 0.00561) and the *COI* gene (Ht = 17, Hs = 0, Hd = 0.743 ± 0.064; π = 0.00621 ± 0.00153). We also estimated genetic divergence including the 4 haplotypes obtained by Nardi et al. [41]. Since Nardi sequences are shorter than ours, we trimmed ours to 432 bp (losing two singleton sites) and run this analysis with this shorter fragment. Therefore, for this matrix we found lower diversity values (N = 12, Ht = 3, Hs = 3, Hd = 0.439 ± 0.025; π = 0.00114 ± 0.00046).

For the *COI* gene the mean *p-distance* between the Atlantic and Pacific clades of *Geotria* was 11.54%, a divergence value that is much larger than the distances between other species of Petromyzontiformes. For example, *p-distance* was 4.67% between *Ichthyomyzon fossor* and *I*. *gagei*, 4.61% between *I*. *gagei* and *I*. *unicuspis*, 0.29% between *Lethentheron appendix* and *L*. *camtschaticum*, 0.12% between *Entosphenus lethophagus* and *E*. *similis*, and 0.04% between *Lampetra planeri* and *L*. *fluviatilis*, with the exception of *Mordacia* where distance between Australian and Chilean species is 19%.

For the *Cyt b* gene, *p-distance* between the Atlantic and Pacific clade of *Geotria* is 16%, was also much larger than the *p*-*distances* between other species pairs of Petromyzontiformes (e.g., 8.21% between *Ichthyomyzon fossor* and *I*. *gagei*, 8.21% *I*. *gagei* and *I*. *unicuspis*, 0.33% between *Lethentheron appendix* and *L*. *camtschaticum*, 0.62% between *Entosphenus lethophagus* and *E*. *similis*, and 0.35% *Lampetra planeri* and *L*. *fluviatilis*), but lower that the distance between *Mordacia* species from Australia and Chile (21%). These values didn’t change when we trimmed the sequences to match those of Nardi et al. [41].

For both genes, the extremely low mean p-distances found between *Lampetra planeri* and *L*. *fluviatilis* and *Entosphenus lethophagus* and *E*. *similis* are consistent with the findings of Lang et al. [44] who suggested that the lack of significant genetic divergence between these species could be attributed to the existence of alternative morphotypes that correspond to parasitic and nonparasitic life history strategies within single species.

**Fig 7:**
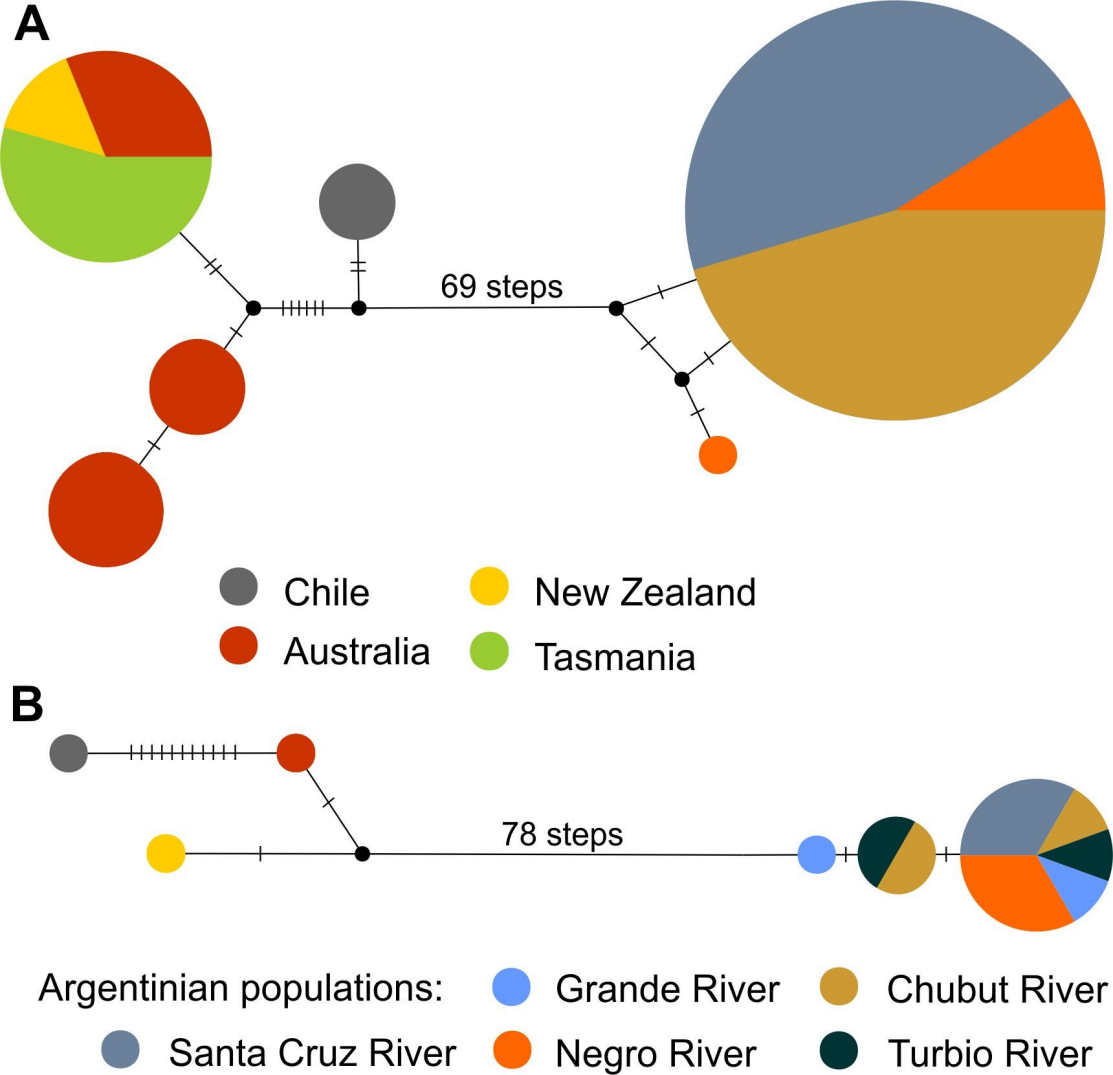
Median joining haplotype network of *Geotria* obtained with the *COI* (A) and *Cyt b* (B) data sets. The circles represent haplotypes, and the circle sizes are proportional to the haplotype frequencies. Mutational steps between haplotypes are indicated along the branches. Circles are colored according to population membership.

The haplotype network revealed the great genetic divergence separating Australasian/ Chilean *Geotria australis* (“Pacific clade”) and Argentinian lampreys (“Atlantic clade”) with more than 69 step mutations in the *COI* gene and more than 78 step mutations in the *Cyt b*. In the COI network Australasia populations shared three common haplotypes diverging by at least 11 steps from the Chilean haplotype. All Argentinian populations shared one single most frequent haplotype with the exception of one distinct haplotype identified in the Negro River. In the *Cyt b* network only two different haplotypes were found, differentiated by 7 steps from the Chilean haplotype. In Argentinian populations, the most frequent haplotype was shared by all populations. Two additional closely related haplotypes were found, one in the Grande River and the other shared by Chubut and Turbio Rivers (Fig 7).

The ABGD analyses for the two data sets (*Cyt b* and *COI*) and the three distance models produced the same results (Table 2). For both markers and the three metrics, initial partitions clustered into 15 haplotypes. Of the 20 reference species included in the analysis 1) *Geotria* from Argentina was distinguished from 2) *Geotria australis* from Chile and Australasia, while 3) *Mordacia precox* and *M*. *mordax* from Australia, 4) *Ichthyomyzon fossor* and *I*. *unicuspis*, 5) *Tetrapleurodon geminis*, *Entosphenus similis* and *E*. *lethophagus*, and 6) *Lethenteron appendix* and *L*. *camtschaticum* were placed in the same clusters. Recursive partitions resulted in 15 (uncorrected p-distance) to 17 clusters (JC69 and K80 with prior intraspecific distances of 0.0010) for *Cyt b*, while for the *COI* fragment recursive partitions ranged from 16 to 22 clusters for the three metrics with prior distances below 0.0050 (Table 2). When recursive partitions recovered 15 clusters, the results were the same as with initial partition. When recursive partitions found 16 clusters, the non-Geotriidae groups were the same as with the initial partition but *Geotria* was subdivided into one cluster from Argentina, one from Chile, and one from Australasia. At 17 clusters one haplotype found in *Geotria* from Argentina was separated from the remaining Argentinian haplotypes. Higher order partitions further subdivided *Geotria* but never discriminated between reference species that clustered together with initial partitions, which could be reflecting that recursive partitions overestimate clusters within *Geotria*, partitioning samples from the same population with two mutations between them.

**Table 2:** Results of ABGD analyses with the Jukes-Cantor (JC69), Kimura (K80), and the uncorrected p-distance (SD) models for the two data sets. Values correspond to the initial and recursive (in parentheses) partitions.

*Prior intraespecific distance (P)*		COI			Cyt b	
	JC69	K80	SD	JC69	K80	SD
*0.0010*	15 (22)	15 (22)	15 (16)	15 (18)	15 (18)	15 (15)
*0.0017*	15 (19)	15 (19)	15 (16)	15 (17)	15 (17)	15 (15)
*0.0028*	15 (18)	15 (18)	15 (16)	15 (15)	15 (15)	15 (15)
*0.0046*	15 (16)	15 (16)	15 (16)	15 (15)	15 (15)	15 (15)
*0.0077*	15 (15)	15 (15)	15 (15)	15 (15)	15 (15)	15 (15)
*0.0129*	15 (15)	15 (15)	15 (15)	15 (15)	15 (15)	15 (15)
*0.0215*	15 (15)	15 (15)	15 (15)	15 (15)	15 (15)	15 (15)
*0.0359*	15 (15)	15 (15)	15 (15)	15 (15)	15 (15)	15 (15)
*0.0599*	15 (15)	15 (15)	15 (15)	15 (15)	15 (15)	15 (15)

## Discussion

### Taxonomic uncertainty within *Geotria*

Since original descriptions of *Geotria* in Argentina between 1868 and 1915, there have been no detailed taxonomic studies of this species, and all subsequent mentions in the literature were based on Nani’s [38] nomenclature, who adopted the synonymy proposed by Maskell [17]. Maskell [17] affirmed that most characters used to define *Geotria* species in the past were simply those distinguishing between sub-adult and adult individuals (e.g., number of lingual teeth, coloration, size and disposition of fins, and development of the gular pouch in males). Following this criteria, since the 1950s all Argentinian lampreys were designated as *Geotria australis*.

Nevertheless, there are two distinct morphological characters that differentiate between Australasia and Argentinian populations. These characters have been reported by many authors along the morphological revisions of *Geotria* [6, 16, 17, 20, 22, 23, 26, 29, 30, 33, 37, 38] and have been confirmed in the individuals examined in this study: published descriptions of *Geotria australis* from Chile and Australasia reported that the second dorsal and the caudal fin are separate and the cloaca is positioned anterior to or under the origin of the second dorsal fin. Whereas in Argentinian specimens the second dorsal and caudal fins are contiguous, connected by a low skin fold and the cloaca is located well posterior to the origin of the second dorsal fin rather than under its origin. Along with the strong genetic differentiation found in this study (see below), morphological differences provide complementary evidence to separate the genus *Geotria* in two distinct species: *G australis* inhabiting Chile and Australasia, and *Geotria macrostoma* (Burmeister 1868) [23], which is the oldest available valid name for identifying Argentinian populations.

### Phylogenetic relationships of *Geotria* from South America and Australasia

The present study has found differences in the mtDNA sequences between *Geotria* from the major Atlantic basins in Argentina (Negro, Chubut and Santa Cruz Rivers–this study, and Turbio and Grande Rivers) and those from Chile and Australasia, which are much greater than expected for populations of the same species. These results indicate that Argentinian populations may represent a distinct species, markedly different from *G*. *australis*. Further, our data agree with those obtained by Nardi et al [41] who recently reported great genetic differentiation between lamprey populations from the Gallegos and Grande Rivers, at the southernmost tip of Patagonia, and *G*. *australis*, concluding that these populations may represent a different species of lamprey unreported for Argentina.

The results of our phylogenetic analysis, based on two mitochondrial genes, showed that South America is inhabited by two *Geotria* clades clearly separated: an “Atlantic” (Argentinian populations) and a “Pacific” clade (Chile and Australasia populations), with strong molecular divergence. Within the Pacific clade, Chilean *Geotria* is placed as a sister group of southern Australia, Tasmania and New Zealand, which clustered together and were more distant to western Australia (Fig 6 and S1 Fig). Our phylogenetic analyses confirm Renaud’s [7] hypothesis that *Geotria* from Argentina might represent a distinct species from *G*. *australis* located in Chile and Australasia. These results also agree with previous studies from Neira et al. [20, 21] who, based on morphological data of ammocoetes, found that Chilean *Geotria* was more closely related to Australasia than to Argentina.

The great divergence in mitochondrial DNA sequences observed for lamprey populations from southern Argentina is similar or even higher than the divergence obtained between species of different genera from the Northern Hemisphere, an observation that led by Nardi et al. [41] to conclude that they may possibly represent a new monotypic genus within Geotriidae. However, in this study, we found that the haplotypes identified by these authors in lampreys collected at the Grande and Turbio (Gallegos) Rivers were genetically identical to the haplotypes we identified in populations from the Santa Cruz, Chubut, and Negro Rivers, in northern Patagonia basins. Argentinian haplotypes clustered together within the “Atlantic” clade and the species delimitation analysis didn’t separate them as different taxa. Therefore, these results indicate that the Argentinian lamprey, *G*. *macrostoma*, constitutes a single species throughout Patagonia, distributed across a broad latitudinal range of at least 15° (from 40°S to 55°S at the 21st century, and from 34°S to 55°S, at least, at the beginning of the 20th century).

### Population structure of *Geotria australis* and *G*. *macrostoma* in South America

**Fig 8:**
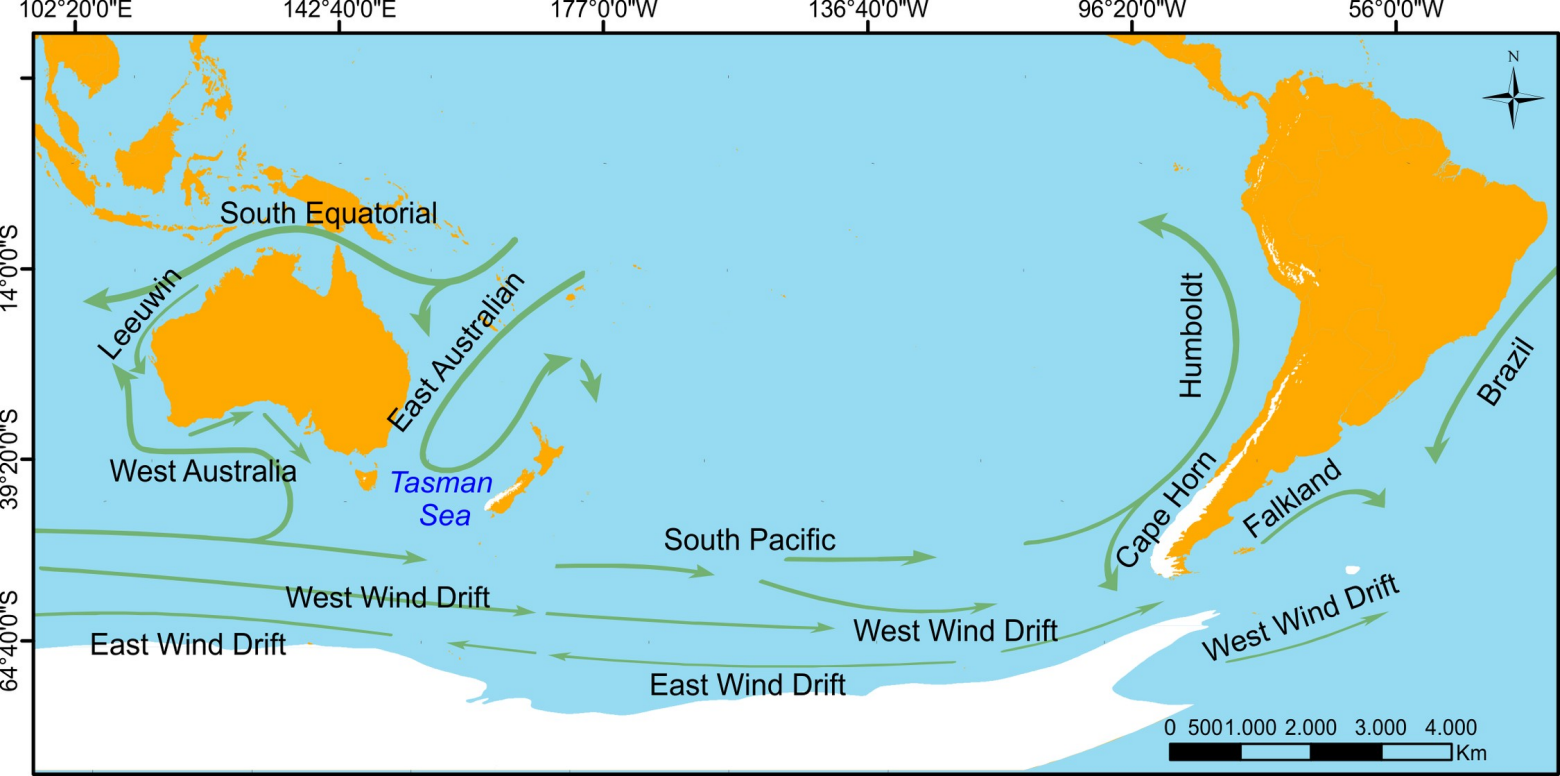
Map of main ocean currents showing ice coverage (white areas) during the Pleistocene glaciations (2.5 mya—10,000 ya).

In this study we recovered high genetic divergence between *Geotria* west and east to the Andes, meanwhile in Argentina, populations spanning across the extra-Andean Patagonian steppe were almost monomorphic, with negligible levels of genetic structuring, a pattern concordant with the phylogeographic patterns documented in several freshwater species of Patagonia [71, 75–78]. Several South American freshwater fish species display deep phylogeographical differences that likely represent the split of Atlantic and Pacific lineages and have been associated with the uplift of the southern Andes (beginning 23 million years ago) and the Pleistocene glaciations (2.5 million years ago—10,000 years ago) [95] (*e*.*g*., *Percichthys trucha*, [77, 78]; *Galaxias maculatus* and *G*. *platei*, [79, 80]; *Trichomycterus areolatus* [81], and *Diplomystes* sp, [82]). The rise of the Andes initially created a permanent barrier to dispersal, separating formerly juxtaposed or connected lineages into distinct Atlantic and Pacific lineages (vicariance). Quaternary glaciations further reinforced the subdivision and structuring of lineages into the separate ice refugia, particularly west of the Andes, where glaciers covered the land from the Andes to the Pacific Ocean (39° to 56°S), during the last glacial maximum (LGM) (18,000–23,000 years ago) [77–86] (Fig 8).

However, on the east side of the Andes, fish species from Atlantic drainages exhibit low phylogeographic structure and divergence [71, 75–78]. This is consistent with our results indicating that populations of *Geotria* from Argentina spanning across the extra-Andean Patagonian steppe were almost monomorphic, with negligible levels of genetic structuring. For many native fish species, high population connectivity across disjunct drainages have been explained as the result of past mixing in the extensive palaeolakes formed during the retreat of the glaciers and dispersal between the interconnected adjacent palaeorivers that formed across the continental shelf during periods of low sea-level [82, 86–89].

The strong divergence between *Geotria* from Argentina and Chile further indicates the lack of exchange/connectivity between adults returning from the sea to spawn into their respective Atlantic and Pacific basins, most likely due to their disjunct distribution and geographic isolation along the coastline of Southern South America. A review of published records indicated that populations of *Geotria macrostoma* in Argentina are distributed across a vast latitudinal range extending from de la Plata River estuary (34°S) to as far south as rivers emptying into the Beagle Channel in Tierra del Fuego (54°S; [41]) and also in the Malvinas (Falkland) [42] and South Georgia Islands [7].

In Chile, however, the range of *Geotria australis* is much narrower, extending from 33°S to 45°S (between Valparaíso and Aysén Regions) [76, 94], although recently it has been also recorded in the Baker River (47°S) (E. Habit, personal communication). Historical records indicated the presence of *G*. *australis* as far south as in rivers flowing into the Strait of Magellan (53°S) and in central Tierra del Fuego [20, 34, 37]. However, based on our results, morphological descriptions of these individuals allow us to identify them as *G*. *macrostoma*. Therefore, despite intensive sampling, at present the species has been declared as “extremely rare or absent” in basins south of 45°S in Chile [76] (Fig 1). The restricted distribution of *G*. *australis* along the Pacific coast of Chile, similar to what have been observed for other species, such as *Mordacia lapicida* and *T*. *aerolatus*, could be a consequence of the long-lasting effects of the loss of suitable riverine habitat for freshwater species, or those with protracted freshwater rearing, during the Last Glacial Maximum, when vast ice sheets covered all but the uppermost reaches of the drainages and converted the headwaters reaches into lentic, lacustrine habitats [81].

Combined with geographic distance, ocean circulation patterns might also explain the population structure between *Geotria* from Chile and Argentina. In fact, Neira et al., [21] and Potter et al. [72] have affirmed that ocean migrating *Geotria* from Argentinian basins utilize hosts that move southwards and eastwards to areas close to the South Georgia Islands during the summer, before migrating back to rivers on the Argentinian coast. Ocean migrating *Geotria* originating from Chilean rivers north of 41°S most likely utilize hosts that move northwards to feed in the sea, a migratory pattern influenced by the prevailing northward Humboldt Current off the Chilean coast (Fig 8).

A similar dispersal scenario has been proposed for *Mordacia lapicida*, present in the northern localities of Chile but absent from rivers of southern Chile and Argentina [73]. Fish dispersal routes around the southern cone of South America and Pacific-Atlantic connectivity have been observed in invading populations of Chinook salmon (*Oncorhynchus tshawytscha*) from Chile and Argentina. It has been proposed that salmon straying from northern locations of Chile are carried further north by the northward Humboldt Current following prey species, in the same way *Mordacia* and *G*. *australis* from northern Chile disperse northward following their host species. In contrast, salmon produced in the fjords of southern Chile, at locations where *Geotria* is apparently absent, are carried away into the Atlantic Ocean, invading Argentinean basins. Such movements are favored by the cold waters of the eastward flowing West Wind Drift and southward by the Cape Horn Current and the Antarctic Circumpolar Current to continue eastward and northward converging into the Malvinas (Falkland) Current (Fig 8). This facilitates southward salmon dispersal from Chilean locations into the Antarctic convergence and into the Patagonian Shelf in the southwestern Atlantic Ocean [74, 75].

### Trans-Pacific dispersal of *Geotria* australis from Chile and Australasia

The genetic relationships recovered by our phylogenetic analysis among Australasian *Geotria* are consistent with both, geographic distance and ocean currents. The marine waters of temperate Australia are dominated by the Leeuwin Current, that originates in northwestern Australia and flows southward and eastward along the west and south coast to reach the Tasman Sea and Southern Ocean, and the East Australia Current that flows southward along the east coast of Australia to the Tasman Sea (Fig 8). Together these currents shape the climate and have considerable influence on marine flora and fauna of Australia [65].

As has been proposed for other marine organisms (such as barnacles; [66]), marine currents could have facilitated dispersal of juvenile *Geotria* from Western to Southern Australia, Tasmania and, subsequently, to New Zealand across the Tasman Sea. Such a pattern is reflected by the weak genetic differentiation among these populations. Since *Geotria* does not exhibit a marine larval phase, it is likely that ocean dispersal in this species relates to the requirements of juvenile feeding. In this regard, the oceanic distribution of individuals could be influenced by the availability and distribution of host species [e.g., 21].

Despite the great distance separating the land masses of the Southern Hemisphere, connectivity between *Geotria* from Australasia and South America is not an unusual finding. Several southern temperate plants and animals that exhibit trans-Pacific similarities has been explained as the result of bidirectional long distance dispersal mediated by the eastward West Wind Drift and the westward East Wind Drift (23 million years ago to present) (Fig 3) [70, 71] or vicariance concordant with the pattern of continental breakup of the supercontinent of Gondwana (between 180 and 35 million years ago), causing the division of an ancestral biota by the increasing opening of the Pacific Ocean [67] and the origin of South America, Africa, and Australasia [68]. Gondwanan forms are, therefore, lineages that are distributed predominantly in the southern continents of South America, Africa—Madagascar, and Australia [67, 68]. For example, freshwater *Galaxias*, and the beech-like trees, *Nothofagus*, present in Australasia and South America, have been regarded as vicariance examples [69]. In fact, vicariance has been more often invoked for explaining trans-Pacific disjunctions of terrestrial and freshwater animal species [69], while long distance dispersal has been more often invoked to explain biotic links between plants, anadromous *Galaxiids* and marine animals [66, 67, 70, 71]. In our case, it is possible that the low genetic divergence and phenotypic similarity between Chilean and Australasian *Geotria* could well be the result of a relatively recent Trans-Pacific long-distance dispersal event during the late Quaternary (last 1 million years), possibly using the coastline of Antarctica as a stepping-stone for dispersal from South America to New Zealand, as has been suggested for some plant taxa and to a lesser extent for animal taxa [70, 71].

### Conservation importance of *G*. *macrostoma* in Argentina

Considering the existence of a second *Geotria* species in the Southern Hemisphere, it becomes critical to undertake biological assessments on the vulnerability of *G*. *macrostoma* to current and expected anthropic impacts. Unlike most of the Northern Hemisphere lampreys assessed at least as vulnerable, the pouched lamprey has been considered as DD (data deficient) by the IUCN [90]. Currently, the documented ecology of *G*. *australis* is based exclusively on Australasian populations [6, 11, 91], and, therefore, may not be representative of *G*. *macrostoma*. Given the genetic and morphological differences found in the present study, it is likely that pouched lamprey from Atlantic and Pacific flowing basins may show different life-history and ecological traits such as spawning habitat selection and timing, migration patterns, and swimming abilities.

In two of the largest basins of Patagonia, the Negro and Chubut, hydro-electric dams, channelization of waterways, water abstraction, and land use modification through agriculture and farming may have caused possible adverse impacts on the distribution, abundance and the population status of *G*. *macrostoma*. In the Santa Cruz River, one of the last large free flowing rivers of Patagonia [50], the pouched lamprey remains unimpacted by human activities but the imminent construction of two high-head hydro-electric dams on the main river channel (70-meter-high Condor Cliff Dam and 40-meter-high La Barrancosa Dam) could severely impact the lamprey population distribution and abundance. A similar pattern could have happened in the upper Negro River where no lampreys have been recorded after dams construction, being albeit present downstream the dams.

Because of their limited swimming ability compared to other migratory fishes, in particular at fish passage systems [92], lampreys are particularly vulnerable to high head dams as they block the migratory corridor and impede access to breeding areas, as well as significantly altering the flow and hydrological regime of the river [13]. Loss of habitat through hydro-electric dam development is thought to be one of the main factors responsible for the decline of pouched lamprey within New Zealand and Australia [10, 13]. The risks posed by the hydro-electric dams on the lamprey population in the Santa Cruz River creates an urgent need to generate baseline information to support their conservation and management, including knowledge of the life cycle, distribution, migratory patterns, habitat use and overall ecological requirements. This knowledge will be critical in understanding the limiting factors and threats to the Santa Cruz River lamprey population and ensure its protection and conservation.

## Conclusions

The present study has confirmed the status of *Geotria macrostoma* in Argentina as a sister species of *Geotria australis*, indicating that the genus *Geotria* is represented in the Southern Hemisphere by two species. Since its original description in 1868, the taxonomic status of *Petromyzon macrostomus* Burmeister, 1868 [23] was questioned by several authors and 36 years after its original description from de la Plata River in Argentina it was synonymized with *G*. *australis* [17]. Our results indicate that the Argentinian pouched lamprey is highly divergent from *G*. *australis* at the molecular level, with marked differences in discrete morphological features. Overall, our data indicate that the Argentinian lamprey, currently found along a broad latitudinal gradient on the south-west Atlantic coast of Patagonia (38° to 54°S), should be assigned to the species *Geotria macrostoma* (Burmeister, 1868) and not to *G*. *australis* Gray, 1851, and must therefore be returned to its earliest valid designation in Argentina. *Geotria macrostoma* can now be considered as an endemism from temperate basins flowing into the Southwestern Atlantic Ocean, with distinct local adaptations and evolutionary potential. It is essential that this distinctiveness is recognized in order to guide future conservation and management actions against imminent and future threats posed by human actions in the major basins of Patagonia. For this, further investigations are needed to assess the distribution, abundance and evolutionary ecology of *G*. *macrostoma* throughout Patagonia and to gather a better understanding of its evolutionary history and phylogenetic relationships with *G*.*australis*.

## Supporting information

S1 Fig Maximum likelihood tree based on the analysis of the *COI* and *Cyt b* mitochondrial genes. Numbers below the nodes indicate bootstrap support values. Name of samples for Argentinian *Geotria* are indicated by the institutional acronym and location (Province) of each sample. Terminal taxa where *COI* and *Cyt b* sequences were concatenated are indicated in bold, terminal taxa represented only by the *COI* fragment are shown in plain font and taxa represented only by the *Cyt b* fragment are shown in grey (see S1 Table).
(TIF)

S1 Table List of all the species, voucher numbers, and GenBank accession numbers of the sequences employed in this study. The asterisk (*) indicates the combination of *COI* and *Cyt b* sequences from the same locality, but different voucher. All samples sequenced in this study are deposited in the Ichthyologic collection of Instituto de Diversidad y Evolución Austral (IDEAus-CONICET), Puerto Madryn, Chubut, Argentina. Acronym CNPICT.
(RTF)
