# Supplementary figures and images for: Revalidation of the Argentinian pouched lamprey Geotria macrostoma (Burmeister, 1868) with molecular and morphological evidence
Source: PLoS One. 2020 May 29;15(5):e0233792. doi: 10.1371/journal.pone.0233792 (PMC7259705; doi:10.1371/journal.pone.0233792)

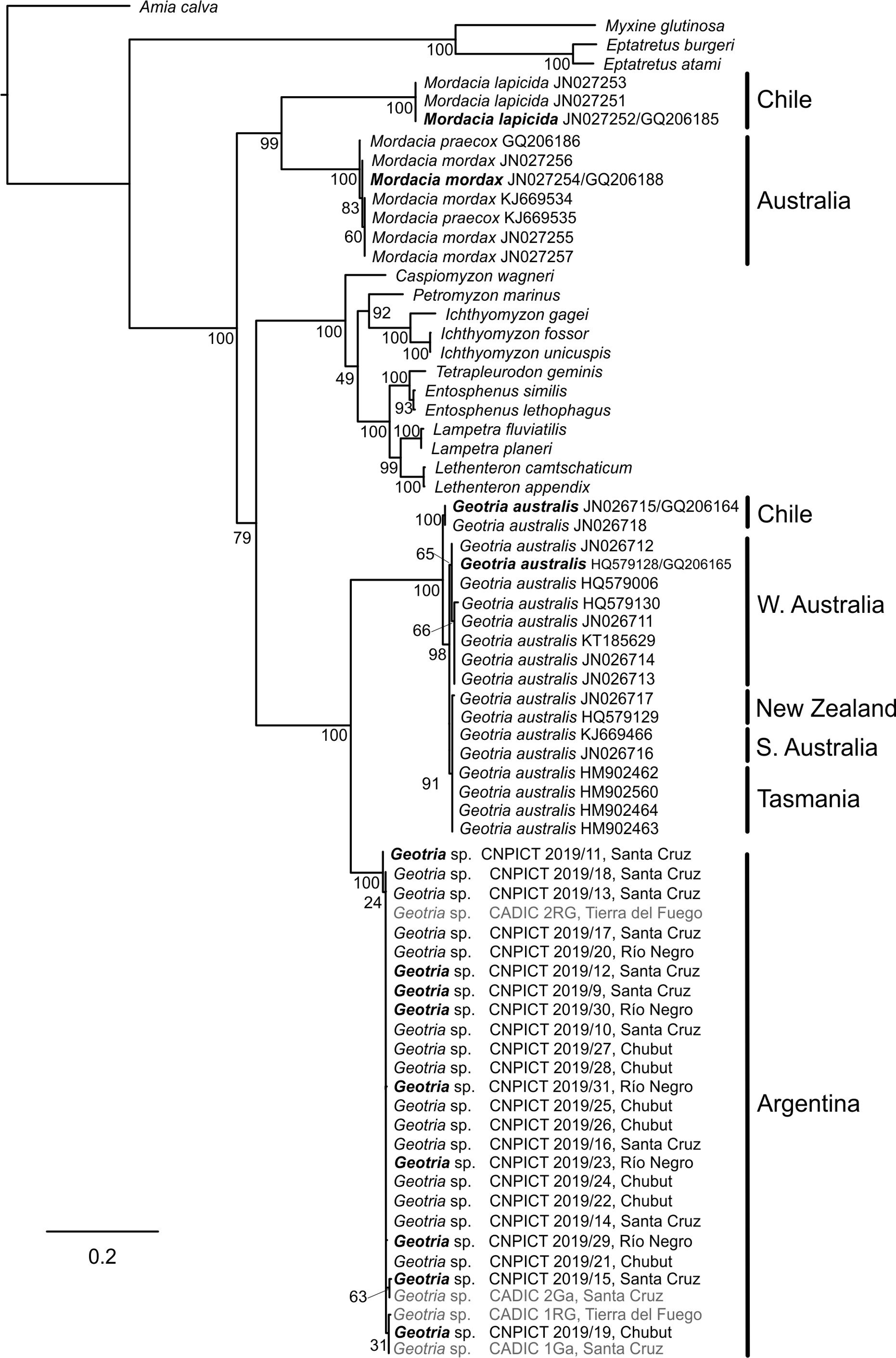

Supplement: S1 Fig — Numbers below the nodes indicate bootstrap support values. Name of samples for Argentinian Geotria are indicated by the institutional acronym and location (Province) of each sample. Terminal taxa where COI and Cyt b sequences were concatenated are indicated in bold, terminal taxa represented only by the COI fragment are shown in plain font and taxa represented only by the Cyt b fragment are shown in grey (see S1 Table). (TIF) [file pone.0233792.s001.tif]
