# Supplementary material for: Revalidation of the Argentinian pouched lamprey Geotria macrostoma (Burmeister, 1868) with molecular and morphological evidence
Source: PLoS One. 2020 May 29;15(5):e0233792. doi: 10.1371/journal.pone.0233792 (PMC7259705; doi:10.1371/journal.pone.0233792)
Supplement: S1 Table — The asterisk (*) indicates the combination of COI and Cyt b sequences from the same locality, but different voucher. All samples sequenced in this study are deposited in the Ichthyologic collection of Instituto de Diversidad y Evolución Austral (IDEAus-CONICET), Puerto Madryn, Chubut, Argentina. Acronym CNPICT. (RTF) [file pone.0233792.s002.rtf]

TABLES1: List of all the species, GenBank accession numbers of the sequences employed in this study, voucher numbers, locality, and source. The asterisk (*) indicates the combination of COI and Cyt b sequences from the same locality, but different voucher. All samples sequenced in this study are deposited in the Ichthyologic collection of Instituto de Diversidad y Evolución Austral (IDEAus-CONICET), Puerto Madryn, Chubut, Argentina. Acronym CNPICT.
Species	COI	Cyt b	Voucher	Locality	Source	
Amia clava	AB042952	CBM-ZF10542	Not reported	[1]	
Caspiomyzon wagneri*	JN024882	GQ206152	NAFF002 / STL801.01	Turkmenistan: Allatepe Bay (Caspian Sea), Balkan Province	[2, 3]	
Entosphenus similis*	JN025331 	GQ206156	NAFF3580 / UAIC12935.01	USA: Klamath River, Oregon	[2, 3]	
Entosphenus lethophagus*	HQ579097	GQ206153	NAFF1929 / UAIC15571.01	USA: Cottonwood Creek, Oregon	[2, 3]	
Eptatretus burgeri	AJ278504	--	Japan: Kingobay, Kagoshima prefecture	[4]	
Eptatretus atami	AP017471	--	Japan: Suruga Bay, Pacific coast 	[5]	
Geotria australis	KT185629	--	New Zealand: Oreti River, Southland	[6]	
Geotria australis*	JN026715	GQ206164	NAFF6311 / STL1260.01	Chile: Andalien River, Biobío Region	[2, 3]	
Geotria australis*	HQ579128	GQ206165	NAFF6057 / UAIC11977.01	Australia: Warren River, Western Australia	[2, 3]	
Geotria australis	KJ669466	--	SAMA:F-FISH84_ML-134	Australia: Goolwa Barrage, The Coorong, SA	Direct submission	
Geotria australis	HM902462	--	BIOUG CAN: BW-A8199	Australia: Tasmania	Direct submission	
Geotria australis	HM902463	--	BIOUG CAN: BW-A8200	Australia: Tasmania	Direct submission	
Geotria australis	HM902464	--	BIOUG CAN: BW-A8201	Australia: Tasmania	Direct submission	
Geotria australis	HM902560	--	BIOUG CAN: BW-A8281	Australia: Tasmania	Direct submission	
Geotria australis	JN026714	--	NAFF025	Australia: Western Australia	[2]	
Geotria australis	JN026713	--	NAFF122	Australia: Western Australia	[2]	
Geotria australis	JN026711	--	NAFF123	Australia: Western Australia	[2]	
Geotria australis	JN026712	--	NAFF124	Australia: Western Australia	[2]	
Geotria australis	HQ579006	--	NAFF125	Australia: Western Australia	[2]	
Geotria australis	HQ579129	--	NAFF6058	Australia: Warren River	[2]	
Geotria australis	HQ579130	--	NAFF6059	Australia: Warren River	[2]	
Geotria australis	JN026717	--	NAFF6061	New Zealand	[2]	
Geotria australis	JN026718	--	NAFF6214	Chile: Pacific Ocean, Andalien River	[2]	
Geotria australis	JN026716	--	NAFF6309	Australia: Victoria, Port Phillip Bay, Tasman Sea, Yarra River	[2]	
Geotria sp.		MK408981	CADIC 1RG	Grande River, Tierra del Fuego Province, Argentina	[15]	
Geotria sp.		MK408982	CADIC 2RG	Grande River, Tierra del Fuego Province, Argentina	[15]	
Geotria sp.		MK408983	CADIC 1Ga	Turbio River, Rio Gallegos Province, Argentina	[15]	
Geotria sp.		MK408984	CADIC 2Ga	Turbio River, Rio Gallegos Province, Argentina	[15]	
Geotria macrostoma	MT478622	MT478645	CNPICT2019/9	Argentina: Santa Cruz River, Santa Cruz Province	This study	
Geotria macrostoma	MT478623	--	CNPICT2019/10	Argentina: Santa Cruz River, Santa Cruz Province	This study	
Geotriamacrostoma	MT478624	MT478646	CNPICT2019/11	Argentina: Santa Cruz River, Santa Cruz Province	This study	
Geotria macrostoma	MT478625	MT478647	CNPICT2019/12	Argentina: Santa Cruz River, Santa Cruz Province	This study	
Geotria macrostoma	MT478626	--	CNPICT2019/13	Argentina: Santa Cruz River, Santa Cruz Province	This study	
Geotria macrostoma	MT478627	--	CNPICT2019/14	Argentina: Santa Cruz River, Santa Cruz Province	This study	
Geotria macrostoma	MT478628	--	CNPICT2019/15	Argentina: Santa Cruz River, Santa Cruz Province	This study	
Geotria macrostoma	MT478629	--	CNPICT2019/16	Argentina: Santa Cruz River, Santa Cruz Province	This study	
Geotria macrostoma	MT478630	--	CNPICT2019/17	Argentina: Santa Cruz River, Santa Cruz Province	This study	
Geotria macrostoma	MT478631	--	CNPICT2019/18	Argentina: Santa Cruz River, Santa Cruz Province	This study	
Geotria macrostoma	MT478632	MT478648	CNPICT2019/19	Argentina: Chubut River, Chubut Province	This study	
Geotria macrostoma	MT478633	--	CNPICT2019/20	Argentina: Chubut River, Chubut Province	This study	
Geotria macrostoma	MT478634	--	CNPICT2019/21	Argentina: Chubut River, Chubut Province	This study	
Geotria macrostoma	MT478635	--	CNPICT2019/22	Argentina: Chubut River, Chubut Province	This study	
Geotria macrostoma	MT478636	MT478649	CNPICT2019/23	Argentina: Chubut River, Chubut Province	This study	
Geotria macrostoma	MT478637	--	CNPICT2019/24	Argentina: Chubut River, Chubut Province	This study	
Geotria macrostoma	MT478638	--	CNPICT2019/25	Argentina: Chubut River, Chubut Province	This study	
Geotria macrostoma	MT478639	--	CNPICT2019/26	Argentina: Chubut River, Chubut Province	This study	
Geotria macrostoma	MT478640	--	CNPICT2019/27	Argentina: Chubut River, Chubut Province	This study	
Geotria macrostoma	MT478641	--	CNPICT2019/28	Argentina: Chubut River, Chubut Province	This study	
Geotria macrostoma	MT478642	MT478650	CNPICT2019/29	Argentina: Negro River, Rio Negro Province	This study	
Geotria macrostoma	MT478643	MT478651	CNPICT2019/30	Argentina: Negro River, Rio Negro Province	This study	
Geotria macrostoma	MT478644	MT478652	CNPICT2019/31	Argentina: Negro River, Rio Negro Province	This study	
Ichthyomyzon fossor	KM267716	--	USA: Canada Creek in Montmorency County, Michigan	[7]	
Ichthyomyzon gagei	KY056640	USI # R2146	USA: Cadron Creek, Arkansas	[8]	
Ichthyomyzon unicuspis	KM267717	--	USA: Peshtigo River, Marinette County, Wisconsin	[7]	
Lampetra fluviatilis	Y18683	--	France: Atlantic Ocean, estuary of the Garonne 	[9]	
Lampetra planeri*	KR477211	GQ206149	-- / STL1118.01	Germany: Kalte Moldau, Bavaria	[3, 10]	
Lethenteron appendix1	KM267719	--	USA: Betsie River, Benzie County, Michigan	[11]	
Lethenteron camtschaticum	KJ866209	--	Russia: Amur River estuary	[12]	
Mixine glutinosa	AJ404477	--	Sweden: Baltic Sea, south	[13]	
Mordacia lapicida*	JN027252	GQ206185	NAFF6211 / STL1260.01	Chile: Andalien River, Biobío Region	[2, 3]	
Mordacia lapicida	JN027253	--	NAFF6212	Chile: Andalien River, Biobío Region	[2]	
Mordacia lapicida	JN027251	--	NAFF6213	Chile: Andalien River, Biobío Region	[2]	
Mordaciamordax*	JN027254	GQ206188	NAFF062 / UAIC15569.01	Australia: Yarra River, Victoria	[2, 3]	
Mordacia praecox	KJ669535	--	isolated CES-358	Australia: Tuross River, NSW	Direct submission	
Mordacia praecox	--	GQ206186	UAIC15570.01	Wallaganaugh River, New South Wales, Australia	[3]	
Petromyzon marinus	U11880	--	USA: Cochecho River, Dover 	[14]	
Tetrapleurodon geminis*	JN028425	GQ206187	NAFF3583 / UAIC15575.01	Mexico: Duero River, Michoacan	[2, 3]	

References
1.	Inoue JG, Miya M, Tsukamoto K, Nishida M. Basal actinopterygian relationships: A mitogenomic perspective on the phylogeny of the “ancient fish”. Mol Phylogen Evol. 2003;26:110-20. doi: 10.1016/S1055-7903(02)00331-7.
2.	April J, Mayden RL, Hanner RH, Bernatchez L. Genetic calibration of species diversity among North America's freshwater fishes. Proc Natl Acad Sci. 2011;108:10602–7. doi: 10.1073/pnas.1016437108.
3.	Lang NJ, Roe KJ, Renaud CB, Gill HS, Potter IC, Freyhof J, et al. Novel relationships among lampreys (Petromyzontiformes) revealed by a taxonomically comprehensive molecular dataset. In: Brown LR, Chase SD, Mesa MG, Beamish RJ, Moyle PB, editors. Biology, Management, and Conservation of Lampreys in North America. 72. Bethesda: American Fisheries Society Symposium; 2009. p. 41-55.
4.	Delarbre C, Gallut C, Barriel V, Janvier P, Gachelin G. Complete mitochondrial DNA of the Hagfish, Eptatretus burgeri: The comparative analysis of mitochondrial DNA sequences strongly supports the Cyclostome monophyly. Mol Phylogen Evol. 2002;22:184-92. doi: 10.1006/mpev.2001.1045. .
5.	Suzuki A, Komata H, Iwashita S, Seto S, Ikeya H, Tabata M, et al. Evolution of the RH gene family in vertebrates revealed by brown hagfish (Eptatretus atami) genome sequences. Mol Phylogen Evol. 2016;107:1-9. doi: 10.1016/j.ympev.2016.10.004.
6.	Ren J, Pu J, Buchinger T, Zhu X, Baker C, Li W. The mitogenomes of the pouched lamprey (Geotria australis) and least brook lamprey (Lampetra aepyptera) with phylogenetic considerations. MITOCHONDRIAL DNA A. 2016;27:3560-2. doi: 10.3109/19401736.2015.1074218.
7.	Ren J, Buchinger T, Pu J, Jia J, Li W. Complete mitochondrial genomes of paired species northern brook lamprey (Ichthyomyzon fossor) and silver lamprey (I. unicuspis). MITOCHONDRIAL DNA A. 2016;27:1862-3. doi: 10.3109/19401736.2014.971261.
8.	Strange RM, V.T. T, Delaney KJ. The mitogenome of the southern Brook Lamprey, Ichthyomyzon gagei (Cyclostomata: Petromyzontidae). MITOCHONDRIAL DNA B. 2016;1:903-4. doi: 10.1080/23802359.2016.1258347.
9.	Delarbre C, Escriva H, Gallut C, Barriel V, Kourilsky P, Janvier P, et al. The complete nucleotide sequence of the mitochondrial DNA of the Agnathan Lampetra fluviatilis: Bearings on the phylogeny of Cyclostomes. Mol Biol Evol. 2000;17:519-29. doi: 10.1093/oxfordjournals.molbev.a026332.
10.	Thalinger B, Oehm J, Mayr H, Obwexer A, Zeisler C, Traugott M. Molecular prey identification in Central European piscivores. Mol Ecol Resour. 2016;16:127-37. doi: 10.1111/1755-0998.12436.
11.	Pu J, Ren J, Zhang Z, Jia L, Buchinger T, Li W. Complete mitochondrial genomes of Korean lamprey (Lethenteronmorii) and American brook lamprey (L. appendix). MITOCHONDRIAL DNA A. 2016;27:1860-1. doi: 10.3109/19401736.2014.971260.
12.	Balakirev ES, Parensky VA, Ayala FJ. Complete mitochondrial genomes of the anadromous and resident forms of the lamprey Lethenteron camtschaticum. MITOCHONDRIAL DNA A. 2016;27:1730-1. doi: 10.3109/19401736.2014.961143.
13.	Delarbre C, Albrekt A, Arnason U, Gachelin G. The complete mitochondrial genome of the Hagfish Myxine glutinosa: Unique features of the Control Region. JMolE. 2002;53:634-41. doi: 10.1007/s002390010250.
14.	Lee WJ, Kocher TD. Complete sequence of a sea lamprey (Petromyzon marinus) mitochondrial genome: early establishment of the vertebrate genome organization. Genetics. 1995;139:873-87.
15.	Nardi CF, Sánchez J, Fernandez DA, Casalinuovo MA, Chalde T. Detection of lamprey in Southernmost South America by environmental DNA (eDNA) and molecular evidence for a new species. Polar Biol. 2020. doi: 10.1007/s00300-020-02640-3.
